# Trait Impulsivity as a Feature of Parkinson's Disease Treatment and Progression

**DOI:** 10.1155/2024/8770997

**Published:** 2024-05-11

**Authors:** Holly Spencer, Ryan S. Anderton

**Affiliations:** School of Health Sciences, University of Notre Dame Australia, Fremantle, WA, Australia

## Abstract

Heightened trait impulsivity in both subclinical and pathological senses is becoming increasingly recognised in Parkinson's disease (PD). Impulsive behaviours and impulse control disorders (ICDs) are a consequence of perturbation to the rewards pathway leading individuals to conduct activities in a repetitive, excessive, and maladaptive fashion. Commonly linked to PD, heightened trait impulsivity has been found to primarily manifest in the forms of hypersexuality, pathological gambling, compulsive shopping, and binge eating, all of which may significantly impact social and financial standing. Subsequent burden to quality of life for both individuals with PD and caregivers are common. Although risk factors and indicators for ICDs in PD are currently lacking, it is recognised that the condition is often precipitated by dopamine replacement therapies, primarily dopamine agonist administration. While this nonmotor symptom is being increasingly diagnosed in PD populations, it remains relatively elusive in comparison to its motor counterparts. Through discussion of impulsivity characteristics, neuroanatomy, and neurochemistry, in addition to reviewing existing research on the potential contributing factors to impulsivity in PD, this review highlights impulsivity as a significant and detrimental PD symptom. Thus, emphasising the imperative need to establish efficacious diagnostic tools and treatments.

## 1. Introduction

Parkinson's disease (PD) is a chronic neurodegenerative condition second only to Alzheimer's disease in global prevalence [1]. Typified by a range of motor symptoms including resting tremor, bradykinesia, rigidity, and reduced postural reflexes, PD also commonly presents with nonmotor symptoms (NMS), often preceding motor signs by many years. Such nonmotor deficits include olfactory, psychiatric, cognitive, and autonomic disturbances [2, 3].

Prodromal cognitive deficits are of particular interest, offering potential insight into disease progression, early diagnosis, and disease-modifying therapies. Notably, heightened trait impulsivity and impulse control disorders (ICDs) are becoming increasingly associated with PD through the administration of dopaminergic medications and disease pathology itself [4, 5]. Recent studies have estimated ICDs to occur in up to 40% of people with PD [6], with males more likely to report multiple impulsive behaviours [7]. Impulsive behaviours reported in people with PD frequently include hypersexuality, pathological gambling, compulsive shopping, and binge eating, possibly leading to financial and social consequences and negatively impacting quality of life (QoL) [5]. Although higher trait impulsivity has been associated with a younger age of PD onset [7], there are no indicators of current or future impulsivity arising. With individuals naturally having varied ranges of impulsivity along the spectrum, it is hypothesised that individuals diagnosed with PD are more likely to have heightened trait impulsivity, possibly resulting in certain individuals falling into the classification of an ICD diagnosis. This review explores the multifaceted nature of impulsivity to provide a greater awareness of this less commonly recognised component of PD treatment and pathology.

## 2. Neuropsychiatric Nonmotor Deficits in PD

The neuropathology underlying neurodegeneration in PD extends to several extranigral regions, thereby compromising dopaminergic, serotonergic, adrenergic, and cholinergic neurotransmission systems [8, 9]. Due to the significance of such systems in modulating processes including cognitive function, emotional processing, circadian rhythm, and reward, dysregulation and neurodegeneration of associated neurons is tightly linked to people with PD experiencing a range of NMS. Cognitive impairment and dementia are frequently reported disorders occurring in both early and late stages of PD. These may occur in conjugation with numerous other psychiatric impairments including depression, anxiety, apathy, and sleep disturbances, which often compound disability and have important implications for mortality [10]. Neuropsychiatric NMS are consequences of the neurodegenerative process and can often complicate the diagnostic process leading to misdiagnoses and mismanagement of disease by both carer and clinician [11]. Depression and anxiety are commonly diagnosed in place of PD when one is presenting with NMS, leading to inflated health-related costs and infrequent referral to neurology specialists [11]. As such, diagnosis of PD can be delayed on average by 1.6 years in comparison to a typical 1.0-year delay in patients presenting with cardinal motor deficits [11]. Thus, early detection of NMS, potentially prior to motor symptom presentation, is imperative. Neuropsychiatric NMS are considered an important cause of morbidity, particularly later in disease progression, and are a significant determinant of patient QoL with recent research emphasising its impact over motor symptoms [10]. There is also significant impact on caregiver burden with increased rates of institutionalisation [12].

Recently, neuropsychiatric disturbances such as increased psychosis and trait impulsivity have gained recognition as neuropsychiatric impairments attributed not only to PD pathology, but interestingly as an adverse effect of dopamine agonist (DAA) use following PD diagnosis. Thus, the recognition and development of suitable treatments for NMS is a critical component in delivering comprehensive and holistic care for PD patients [13].

## 3. Trait Impulsivity in PD

Impulsivity is a multifaceted construct underpinned by cognitive and motor systems. In the general population, individuals vary in their natural tendency to behave impulsively, known as trait impulsivity. Cognitively, high trait impulsivity includes maladaptive decision-making [14], increased risk taking [15], intolerance for delayed rewards (temporal discounting) [16], and the inability to adjust behaviour based on environmental feedback (reversal learning) [17]. Motorically, high trait impulsivity involves diminished response inhibition, or the inability to inhibit a prepotent motor response [18]. High trait impulsivity is a risk factor for the development of severe neuropsychiatric disorders such as substance abuse disorder [19], borderline personality disorder [20], and ICDs [21]. Moreover, current conceptions of impulsivity support a dimensional approach, in which personality and pathological impulsivity represent dimensions of the same condition. Indeed, as a neuropsychiatric complication of PD, impulsivity ranges in severity from mild trait impulsivity changes to severe, clinically identifiable ICDs [22–24]. Recent studies have suggested the prevalence of trait impulsivity in the PD population is associated with increased ICD diagnosis.

With focus on subclinical changes to trait impulsivity, recent studies have revealed that individuals diagnosed with PD and lacking an ICD diagnosis behaved more impulsively than healthy controls [22, 25–27]. Furthermore, higher trait impulsivity has been reported in people with PD with comorbid ICDs in contrast to ICD-free PD patients [28, 29]. Consequently, high trait impulsivity is postulated as a risk factor for ICD development in PD [24]. Despite this knowledge, a significant gap remains regarding the epidemiology and pathophysiology of trait impulsivity in PD, highlighting the necessity for further studies [22, 30, 31]. Such investigations would provide significant information for the ∼85% of PD patients who do not fit the ICD diagnostic criteria but may experience subsyndromal impulsivity symptoms. Notably, such investigations may further elucidate the complex nature of impulsivity initiation and progression, leading to the identification of impulsivity indicators, comprehensive diagnostic tests, and potentially, treatment of such behaviour with appropriate intervention.

### 3.1. Measuring Trait Impulsivity

As impulsivity may encompass both cognitive and motor components, multiple behavioural tasks and self-report questionnaires are utilised in a clinical and diagnostic capacity. Behavioural tasks treat impulsivity as a unitary construct, objectively measuring its cognitive or motor elements. Common cognitive impulsivity tasks assess tolerance of delayed reward (e.g., temporal discounting task) [32], risk taking (e.g., probabilistic discounting task) [33], attention (e.g., continuous performance task) [34], and decision-making (e.g., Iowa gambling task and Wisconsin card sorting task) [35, 36]. Motor impulsivity tasks measure premature responding (e.g., five-choice serial reaction time task and go/no-go task) [37] or response inhibition (e.g., stop-signal reaction time task) [38]. Contrastingly, self-report questionnaires treat trait impulsivity as a multifaceted construct, simultaneously assessing both cognitive and motor elements [39]. The most widely used questionnaire is the Barratt Impulsiveness Scale (BIS-11) which yields scores of total impulsivity, with higher scores indicative of greater impulsivity [40, 41]. Furthermore, through factor analysis, the BIS-11 accounts for second- and first-order subdomains of the trait [41]. Although the total score gained from the BIS-11 is recognised as a reliable indicator of impulsivity, subsequent analyses of this diagnostic test suggests that it does not assess the three impulsivity components of attentional impulsivity, motor impulsiveness, and nonplanning impulsiveness as originally detailed by Barratt [42]. Thus, misinterpretation of impulsivity characterisation, a component which may provide critical information to the patient in addition to the overall BIS-11 score, may occur [42].

With such failings in mind, the Questionnaire for Impulsive-Compulsive Disorders in Parkinson's Disease (QUIP) was designed with the hope of providing a comprehensive diagnostic test in the context of PD [43]. This self-administered screening questionnaire is significant for the identification of individuals who may require a follow-up diagnostic interview, or further monitoring of early recognition of symptoms as they become clinically significant [44]. Although subsyndromal ICD symptoms have been notoriously difficult to detect, the QUIP detected with 100% accuracy in the presence of an ICD in confirmed comorbid PD-ICD populations, in addition to revealing a positive QUIP result in 40% of undiagnosed individuals [43]. Hence, such patient-administered early screening tools may aid in better characterising trait impulsivity that ranges between subclinical presentations to an ICD diagnosis.

Although both behavioural tasks and self-report questionnaires such as the BIS-11 and QUIP are recommended for the detection of trait impulsivity alterations [45] at present, indicators of heightened trait impulsivity are lacking, with DAA use being the primary risk factor suspect. While male sex, no history of smoking, following secondary education, and more severe course of illness have been postulated as possible players, various studies are contradictory and often significance is lacking [31]. Recent studies are also suggestive of REM sleep behaviour disorders (RBDs) and sleep disturbances presenting an identifying factor for future ICD development [46, 47]. Currently, classified as a prodromal stage of alpha-synucleinopathies, RBDs are also proposed to cause a more severe impairment in the mesocorticolimbic pathway, culminating in impairment of reward processing [47].

### 3.2. Impulse Control Disorders

Recognising pathological alterations to trait impulsivity is made complicated due to its variety of presentations. Broadly, the four major ICDs defined are hypersexuality, pathological gambling, compulsive shopping, and binge eating [48]. Individuals with ICD's perform such pleasurable behaviours repetitively, excessively and compulsively to the point of disruption to daily life and harm to others [5]. Individuals generally report being psychologically driven to engage in such behaviours as they are considered to be rewarding, implicating dysregulation of the rewards pathway and multiple neurotransmission systems [9]. People with PD displaying comorbid ICDs, experience drastic social, financial, and legal complications, reporting gambling debts, marriage breakdown, and loss of employment [49, 50]. Furthermore, ICDs are an independent predictor of reduced QoL, particularly regarding emotional wellbeing, and also correlate with greater functional impairment in daily living activities and increased caregiver burden [24, 51, 52]. For example, people with PD exhibiting high trait impulsivity self-report has significantly poorer QoL, with PDQ-39 scores approximately 2-fold higher compared to patients experiencing low trait impulsivity [37].

People with PD may also exhibit ICD-related behaviours that share ICD biology but have contrasting clinical presentations. For instance, repetitive purposeless behaviours (punding), hobbyism, excessive aimless wandering (walkabout), and hoarding and compulsive medication use including dopamine dysregulation syndrome (DDS) [53]. While rare in the general population at 0–0.25%, such behaviours appear in approximately 14% of PD patients [54–56], generally arising subsequent to dopaminergic replacement therapy (DRT) initiation [57, 58]. The first line of treatment following ICD diagnosis involves optimising dopaminergic medications and specifically removing patients from DAA treatment [59]. However, this can result in withdrawal symptoms or the return of motor symptoms. Cognitive behavioural therapy has indicated encouraging results in reducing ICD symptoms [60]. However, its efficacy is reliant on patient self-awareness of disruptive behaviours. As a result, treating both motor symptoms and heightened trait impulsivity simultaneously remains a challenging front.

ICDs have been documented in a portion of people with PD across various studies. With a sample of 3,090 individuals with PD, the DOMINION study reported at least one ICD in 13.6% of patients [56]. This group encompassed 5% gambling, 3.5% compulsive sexual behaviour, 5.7% compulsive buying, and 4.3% binge-eating disorder [56]. Notably, 3.9% of the test group had two or more ICDs [56]. Several studies have acknowledged that the frequency of ICDs may extend beyond these figures, with many patients under-reporting impulsive behaviours due to embarrassment, lack of symptom awareness, and irregular clinical screening [9, 44, 56, 61]. More recent investigation of smaller PD cohorts has established that ICDs may in fact affect up to 40% of people with PD, providing novel evidence that ICDs are considerably under-reported [6]. This high prevalence indicates the necessity for clinicians to enquire as to whether patients and family members' may have identified compulsive behaviours in order to uncover evidence of impulsivity as prematurely as possible.

## 4. Neurobiology of Trait Impulsivity in PD

### 4.1. Neuroanatomy of Trait Impulsivity

Impulsive desire must be tightly self-regulated depending on circumstance, motivation, and emotion, involving an array of neuronal populations. Key players for the modulation of trait impulsivity include the striatum of the basal ganglia and prefrontal cortex, both with opposing yet complimentary functions. The striatum promotes impulsive choices and actions, while the prefrontal cortex and hippocampus permit goal-directed inhibition of such behaviour [62]. Interestingly, activation of these brain regions differs in an age-related fashion, in addition to influences from personality, underlying neuropsychiatric conditions, medications, and neurodegenerative diseases such as PD, resulting in a spectrum of trait impulsivity [56, 63].

The dorsal striatum controls action selection and stimulus-response learning [64]. It is also an integral locus for response inhibition and choice impulsivity. Following disruption to the striatum, impulsivity is adjusted accordingly. Induction of dorso-striatal excitotoxic lesions in rodents slowed responses on the stop-signal reaction time task [65]. Similarly, dopamine D2 receptor antagonism resulted in increased trait impulsivity as a consequence of reduced inhibitory postsynaptic potential generation through the D2 receptor [65]. In humans, reduced D2-receptor binding potential in the dorsal, but not ventral striatum had the same effect [66]. Moreover, on a temporal discounting task, dopaminergic depletion in the dorsolateral striatum produced a steep, impulsive discounting of a delayed reward [67].

Comparably, the ventral striatum consisting of the olfactory tubercle and nucleus accumbens (NAcb) controls motivation, reward processing, and various components of impulsivity [68]. In both rodents and humans, reduced ventral striatal D2/D3-receptor availability and dopamine release was linked to increased trait impulsivity [69, 70], whilst excitotoxic lesions to the NAcb core increased the tendency of rodents to discount delayed rewards [71, 72]. Notably, an apparent opposing modulation exists between the D2/D3 receptors of the NAcb core and shell subregions. Injection of a D2/D3 receptor antagonist into the NAcb shell of highly impulsive rats significantly increased the level of impulsivity as measured by the 5-choice reaction time (5-CSRT) test of sustained visual attention, whilst injection into the NAcb core reduced impulsivity [73]. Regarding D2 receptor agonists, results are ambiguous as impulsivity is either unchanged or only increases in animals whose trait impulsivity is naturally higher [73, 74]. Thus, it is apparent that the action of dopamine and its corresponding receptors show variation according to the brain region and interindividual differences in underlying impulsivity. As the striatum is closely linked to impulsive responding, a regulatory region in the prefrontal cortex and hippocampus is essential for healthy control of impulsive behaviour.

Prefrontal areas and the hippocampus exhibit control of the impulsive drive promoted by the striatum. Frontostriatal loops controlling distinct components of trait impulsivity originate from broad frontal regions [75]. The medial orbitofrontal cortex, anterior cingulate area, and ventromedial prefrontal cortex are linked to emotional decision-making and risk impulsivity, often assessed by probabilistic discounting tasks. Specifically, the medial orbitofrontal cortex is necessary for the valuation of rewards as people with damage to this area prefer immediate, smaller rewards over larger, delayed ones [76]. Furthermore, small orbitofrontal cortical volumes correlated with higher BIS-11 scores, while damage to this region correlates to increased gambling habits [77, 78]. In relation to the anterior cingulate area, decreased gray matter volume has been observed in impulsive populations [78, 79]. A significant component of impulsivity involves the control of executive functions including behavioural planning and goal selection, generally regulated by the dorsolateral prefrontal cortex [80]. Functional MRI has revealed increased activation of the dorsolateral prefrontal cortex when participants select a delayed reward [81, 82] or exercised self-control over food consumption [83]. As response inhibition is a critical component of organised cognitive and behavioural responses, deficient inhibitory processes exhibited in high trait impulsivity suggest insufficient control by the nearby inferior frontal gyrus and prefrontal motor regions [84–87]. Thus, a plethora of prefrontal areas have been implicated in the control of impulsive behaviour, in addition to the hippocampus via the more recently recognised hypothalamus-hippocampus circuitry in which perturbance of this pathway in either direction results in increased impulsivity [88]. In addition, reduced hippocampal volume or cell loss has been observed in highly impulsive populations further supporting its critical role in impulse regulation [89–91]. In each of these neuroanatomical regions, multiple neurotransmission systems are functional in which their dysregulation has been directly correlated to increased trait impulsivity.

### 4.2. Neurochemistry of Trait Impulsivity in PD

Activities in the frontostriatal networks mentioned above is modulated by the ascending monoaminergic systems [92, 93], specifically dopamine from the ventral tegmental area [94], serotonin from the dorsal and medial raphe nuclei [95], and noradrenaline from the locus coeruleus [96]. It should also be noted that non-monoaminergic transmission including GABA, glutamate, and endogenous opioids also modulate trait impulsivity [97]; however such systems are outside the scope of this review.

Changes to impulsivity have been observed when alterations occur in at least one of these three monoaminergic systems. Furthermore, altered dopamine concentrations or dopamine receptor activity in the impulsivity circuitry is linked to impulsive behaviour [66, 69, 70, 98–101], as it is hypothesised that individuals scoring higher on impulsivity measures have their pathological behaviour rooted in a hypersensitive dopamine reward system [102]. To compound this effect, striking clinical evidence exists for the development of ICDs when dopamine receptor agonists are used in the treatment of PD. This can potentially supplement dopamine in the degenerating substantia nigra, whilst overstimulating relatively unaffected reward regions of the brain [53].

In addition to dopamine, serotonin is influential in impulsivity. The depletion of serotonin in both rodents and humans increased impulsive premature responding [103, 104], with low brain serotonin concentrations being observed in highly impulsive suicide attempters [105, 106]. Serotonin hypofunction has also been purported as a predisposing biochemical trait for impulsive aggression [107]. Furthermore, not only is serotonin dysregulation sufficient to increase trait impulsivity, but impairment of the serotonin system is known to cause dysregulation of dopamine neurotransmission, compounding the effect on impulse responses [108].

Although the role of noradrenaline in ICD development in PD has not been studied, animal models indicate increased noradrenergic signaling improving impulse control [109, 110]. Notably, the mechanism of this action is currently unknown and likely dependent on the impulsive behaviour subtype. However, atomoxetine, a selective noradrenaline reuptake inhibitor has profound anti-impulsivity effects including stop-signal reaction time, delay discounting of reward, and the 5-CSRT test in rodents [111]. Hence, this drug has shown efficacy in the treatment of ADHD [112], which is characterised by low noradrenergic function [113]. Furthermore, low concentration of the noradrenergic transporter in the hippocampus and orbitofrontal cortex has been linked to higher BIS-11 scores in healthy controls [114]. Taken together, it is clear that dopaminergic, serotoninergic, and noradrenergic transmission tightly regulates the impulsivity circuitry, with disruption to these systems via an array of influential factors resulting in both heightened and potentially pathological impulsivity.

## 5. Factors Contributing to Trait Impulsivity in Parkinson's Disease

Although specific determinants of impulsivity in PD remain uncertain, a myriad of individual patient factors are likely to contribute to levels of impulsivity. The use of dopaminergic medications has emerged as a potential risk factor for increased trait impulsivity and the development of ICDs, in addition to the influence of PD pathology itself. Given the small number of studies regarding the development of pathological impulsivity in PD, evidence for the development ICDs in the context of PD will also be explored.

### 5.1. Dopaminergic Medications

Dopamine agonists, a common treatment used to address the motor effects of PD, have been hypothesised as influential factors in the increased prevalence of subsyndromal impulsivity symptoms and ICD development in PD. Studies are indicative of alterations to trait impulsivity occurring in a dose dependent manner, with this premise further supported by studies revealing reductions in compulsive behaviour subsequent to DAA dose reductions [115, 116]. A considerable body of evidence exists to support the notion that DAAs provoke the development of ICDs. The DOMINION study identified that DAA use was associated with a 2-3.5-fold increased risk of developing an ICD [56]. Specifically, ICDs were present in 14.0% of PD patients utilising DAAs without levodopa, in comparison to 7.2% of patients treated with levodopa alone, and 1.7% of PD patients not being treated with either a DAA or levodopa [56]. Furthermore, concurrent treatment with both DAA and levodopa significantly compounded the chance of an ICD being present at 17.7%, approximately 50% greater than DAA use alone [56]. Other studies have identified that up to 94% of PD patients demonstrating compulsive behaviours were prescribed concurrent levodopa therapy, further indicating the role of levodopa as an accessory to ICD development [9, 117].

Although levodopa and other alternative medications such as amantadine are associated with ICD development, findings from the DOMINION study concluded that the odds ratio was nearly twice as high for DAAs than levodopa [56]. Instead, levodopa is more likely to cause DDS and compulsive behaviours such as punding over ICDs, likely due to differing receptor specificities of DAAs and levodopa, with DAAs stimulating reward pathways more avidly than levodopa-derived dopamine [50]. Not only have DAAs been tied to ICD development in PD, but an identical pattern of aberrant behaviour has been identified in subjects prescribed DAAs for the treatment of restless leg syndrome (RLS), hyperprolactinemia, and fibromyalgia [118, 119]. It has been reported that a fraction of RLS patients treated with DAAs developed ICDs, with frequencies of 17% for any ICD, 9% for compulsive shopping, 5% for pathological gambling, 11% for compulsive eating, 3% for hypersexuality, and 7% for punding [118]. These findings are comparable with frequencies described in PD patients taking DAAs, corroborating the indication that DAA use, rather than disease type, influence the onset of ICDs.

Specifically, the class of DAA may also be influential in ICD development. Pramipexole and ropinirole, the two most commonly prescribed DAAs, have the greatest association with increased ICD frequency in PD, RLS, and fibromyalgia [6, 56, 119]. According to a range of cross-sectional and longitudinal studies, this phenomenon is potentially associated with the greater specificity and preferential affinity of pramipexole and ropinirole for D3 receptors in comparison to alternative DAAs [27, 56–58, 120–123]. As a result, DAAs such as rotigotine, bromocriptine, and apomorphine have significantly lower ICD frequencies [6, 121]. Alternative studies suggest that DAA subtype holds no significant association and instead other demographic and clinical variables compound presentation [124]. Despite such conflicting results, there is a clear causal relationship between DAA use and ICD development.

Dopamine overdose is hypothesised to mediate the association between DAA use and severe impulsivity, although the mechanism is not completely clear. In early stages of PD, dopaminergic depletion is restricted to the dorsal striatum via nigrostriatal death, while dopaminergic input to the ventral striatum from the ventral tegmental area is relatively maintained [125]. Thus, the introduction of DAA may correct dopaminergic signaling in the deprived dorsal striatum, whilst also overstimulating the ventral striatum, manifesting in severe deficits of impulse control [53, 126]. This is especially true of D3-specific agonists, given the high density of dopamine D3-receptors in the ventral striatum [127]. It has additionally been recognised that prolonged exposure to DRTs, especially levodopa, results in sensitised ventral striatal dopamine transmission with enhanced levodopa-induced dopamine release [128]. As a result of heightened dopamine transmission in the rewards circuitry, DDS positive subjects reported “wanting” as opposed to “liking” levodopa, with amplified psychomotor activation, including punding [128]. Thus, chronic DRT is proposed to further disrupt reward circuitry thereby underlying an increase in repetitive behaviours.

Whilst the high affinity of frequently prescribed DAAs for D3 receptors in the striatal limbic system appears to represent the prevailing argument for the relationship between DAA use and ICD onset, evidence exists for the effects of DAA-induced D2/3 receptor internalisation upon prolonged agonist exposure and hyperdopaminergic state [129, 130]. Specifically, loss of dopamine receptor density and reduced D2/3 nondisplaceable binding potential in both the ventral and dorsal striatum have been correlated to heightened choice impulsivity in rats and increased self-reported ICD symptoms in humans. This is due to the function of midbrain D2/3 receptors in inhibition and their inverse relationship with impulsivity [129, 131]. Furthermore, although DAAs such as pramipexole and ropinirole are D2 receptor subtype specific, to some extent, D1 receptors are also stimulated, leading to an array of undesired side effects due to the wide distribution of these receptors throughout the brain [132]. Contrastingly, the only study into clinical and demographic predictors of trait impulsivity in PD concluded DAA use was not a significant risk factor [31]. However, this study did not account for dosage or DAA subtype, classifying sufferers simply as DAA positive or negative. Seemingly, the interaction of DAA administration and ICD development is complex and requires further elucidation.

### 5.2. Parkinson's Disease Pathology

Although dopaminergic medication use is robustly linked to severe impulsivity symptoms in PD, studies involving drug-naïve and drug-deprived PD patients suggest the disease process itself increases susceptibility to exacerbated impulsivity. Specifically, PD patients responded impulsively on a cognitive test both on and off dopaminergic medication [26]. While this could be due to a short withdrawal period of medication, similar results were found using drug-naïve PD patients [4], indicating heightened impulsivity may be related to depleted nigrostriatal dopamine. A preclinical model of PD featuring bilateral nigrostriatal denervation revealed greater dopamine release in the nucleus accumbens [133], an important driver of impulsivity. In addition, a PET study of untreated PD patients showed reduced binding at dopamine D3-receptors in the ventral striatum [134], which is linked with the occurrence and severity of ICD behaviours [135]. Extranigrally, neurodegeneration of the locus coeruleus and raphe nuclei in PD dysregulates noradrenaline and serotonin [136] which, as discussed, are intimately linked with trait impulsivity. It is also known that in advanced PD disease pathology extends to the neocortex [8]. A recent neuroimaging study highlighted that neocortical thinning in frontal and cingulate areas correlates with high trait impulsivity in PD as prefrontal regions play a critical role in inhibition of impulsive striatal behaviour [30]. Taken together, this data suggest that the pathology of PD itself via neurotransmitter dysregulation and degeneration of both dopaminergic and serotonergic axons may be influential in exacerbating impulsivity [137–139].

## 6. Conclusion

Alterations to trait impulsivity and the development of ICDs in PD are becoming increasingly recognised as a debilitating component of this neurodegenerative condition. Primarily in the forms of hypersexuality, pathological gambling, compulsive shopping, and binge eating, heightened trait impulsivity manifests as uncontrollable behaviours and was carried out despite negative implications. Although dopaminergic medications such as DAAs and levodopa are effective in managing the cardinal motor signs of PD, recent research is indicative of their insidious effects on neuropsychiatric disturbances such as heightened impulsivity. Despite this, minimal studies have explored the risk factors, indicators, and potential modes of treatment for PD-related heightened trait impulsivity. Although PD-related ICDs are difficult to manage and treatments in this field are lacking, early screening for neuropsychiatric fluctuations in addition to education of patients, relatives, and caregivers regarding the early signs of impulsive behaviours is critical. Despite the contribution of anti-Parkinsonian medications to such behaviours and the complex and competing underlying PD pathogenesis, studies have considered the potential treatment approaches available to patients [140]. Appropriately, pharmaceutical, and nonpharmaceutical interventions can ensue, with potential adjustments to dopaminergic medication plans in order to protect this aspect of QoL in PD patients.

## References

[1] Dorsey E. R., Sherer T., Okun M. S., Bloem B. R. (2018). The emerging evidence of the Parkinson pandemic. *Journal of Parkinson’s Disease*.

[2] Jankovic J. (2008). Parkinson’s disease: clinical features and diagnosis. *Journal of Neurology, Neurosurgery & Psychiatry*.

[3] Pellicano C., Benincasa D., Pisani V., Buttarelli F. R., Giovannelli M., Pontieri F. E. (2007). Prodromal non-motor symptoms of Parkinson’s disease. *Neuropsychiatric Disease and Treatment*.

[4] Al-Khaled M., Heldmann M., Bolstorff I., Hagenah J., Münte T. F. (2015). Intertemporal choice in Parkinson’s disease and restless legs syndrome. *Parkinsonism & Related Disorders*.

[5] Weintraub D., David A. S., Evans A. H., Grant J. E., Stacy M. (2015). Clinical spectrum of impulse control disorders in Parkinson’s disease. *Movement Disorders*.

[6] Garcia-Ruiz P. J., Martinez Castrillo J. C., Alonso-Canovas A. (2014). Impulse control disorder in patients with Parkinson’s disease under dopamine agonist therapy: a multicentre study. *Journal of Neurology, Neurosurgery & Psychiatry*.

[7] Abosch A., Gupte A., Eberly L. E., Tuite P. J., Nance M., Grant J. E. (2011). Impulsive behavior and associated clinical variables in Parkinson’s disease. *Psychosomatics*.

[8] Braak H., Tredici K. D., Rüb U., De Vos R. A., Jansen Steur E. N., Braak E. (2003). Staging of brain pathology related to sporadic Parkinson’s disease. *Neurobiology of Aging*.

[9] Hassan A., Bower J. H., Kumar N. (2011). Dopamine agonist-triggered pathological behaviors: surveillance in the PD clinic reveals high frequencies. *Parkinsonism & Related Disorders*.

[10] Liu W. M., Lin R. J., Yu R. L., Tai C. H., Lin C. H., Wu R. M. (2015). The impact of nonmotor symptoms on quality of life in patients with Parkinson’s disease in Taiwan. *Neuropsychiatric Disease and Treatment*.

[11] O’Sullivan S. S., Williams D. R., Gallagher D. A., Massey L. A., Silveira-Moriyama L., Lees A. J. (2008). Nonmotor symptoms as presenting complaints in Parkinson’s disease: a clinicopathological study. *Movement Disorders*.

[12] Prakash K. G., Bannur B. M., Chavan M. D., Saniya K., Sailesh K. S., Rajagopalan A. (2016). Neuroanatomical changes in Parkinson’s disease in relation to cognition: an update. *Journal of Advanced Pharmaceutical Technology & Research*.

[13] Martinez-Martin P., Rodriguez-Blazquez C., Kurtis M. M., Chaudhuri K. R. (2011). The impact of non-motor symptoms on health-related quality of life of patients with Parkinson’s disease. *Movement Disorders*.

[14] Bechara A., Damasio H., Damasio A. R. (2000). Emotion, decision making and the orbitofrontal cortex. *Cerebral Cortex*.

[15] Stanford M. S., Greve K. W., Boudreaux J. K., Mathias C. W., L Brumbelow J. (1996). Impulsiveness and risk-taking behavior: comparison of high-school and college students using the Barratt Impulsiveness Scale. *Personality and Individual Differences*.

[16] Wittmann M., Paulus M. P. (2008). Decision making, impulsivity and time perception. *Trends in Cognitive Sciences*.

[17] Fellows L. K., Farah M. J. (2004). Different underlying impairments in decision-making following ventromedial and dorsolateral frontal lobe damage in humans. *Cerebral Cortex*.

[18] Antonelli F., Ray N., Strafella A. P. (2011). Impulsivity and Parkinson’s disease: more than just disinhibition. *Journal of Neurological Sciences*.

[19] Mitchell M. R., Potenza M. N. (2014). Addictions and personality traits: impulsivity and related constructs. *Current behavioral neuroscience reports*.

[20] Casillas A., Clark L. A. (2002). Dependency, impulsivity, and self-harm: traits hypothesized to underlie the association between cluster B personality and substance use disorders. *Journal of Personality Disorders*.

[21] Houeto J.-L., Magnard R., Dalley J. W., Belin D., Carnicella S. (2016). Trait impulsivity and anhedonia: two gateways for the development of impulse control disorders in Parkinson’s disease?. *Frontiers in Psychiatry*.

[22] Nombela C., Rittman T., Robbins T. W., Rowe J. B. (2014). Multiple modes of impulsivity in Parkinson’s disease. *PLoS One*.

[23] Phu A. L., Xu Z., Brakoulias V. (2014). Effect of impulse control disorders on disability and quality of life in Parkinson’s disease patients. *Journal of Clinical Neuroscience*.

[24] Voon V., Sohr M., Lang A. E. (2011). Impulse control disorders in Parkinson disease: a multicenter case–control study. *Annals of Neurology*.

[25] Gauggel S., Rieger M., Feghoff T. A. (2004). Inhibition of ongoing responses in patients with Parkinson’s disease. *Journal of Neurology, Neurosurgery & Psychiatry*.

[26] Milenkova M., Mohammadi B., Kollewe K. (2011). Intertemporal choice in Parkinson’s disease. *Movement Disorders*.

[27] Obeso I., Wilkinson L., Casabona E. (2011). Deficits in inhibitory control and conflict resolution on cognitive and motor tasks in Parkinson’s disease. *Experimental Brain Research*.

[28] Housden C. R., O’sullivan S. S., Joyce E. M., Lees A. J., Roiser J. P. (2010). Intact reward learning but elevated delay discounting in Parkinson’s disease patients with impulsive-compulsive spectrum behaviors. *Neuropsychopharmacology*.

[29] Voon V., Reynolds B., Brezing C. (2010). Impulsive choice and response in dopamine agonist-related impulse control behaviors. *Psychopharmacology*.

[30] Kubera K. M., Schmitgen M. M., Nagel S. (2019). A search for cortical correlates of trait impulsivity in Parkinson´s disease. *Behavioural Brain Research*.

[31] Riley M., Bakeberg M., Byrnes M. (2018). Demographic and clinical predictors of trait impulsivity in Parkinson’s disease patients. *Parkinson’s Disease*.

[32] Ainslie G. (1975). Specious reward: a behavioral theory of impulsiveness and impulse control. *Psychological Bulletin*.

[33] Green L., Myerson J. (2013). How many impulsivities? A discounting perspective. *Journal of the Experimental Analysis of Behavior*.

[34] Song D.-H., Jhung K., Song J., Cheon K.-A. (2011). The 1287 G/A polymorphism of the norepinephrine transporter gene (NET) is involved in commission errors in Korean children with attention deficit hyperactivity disorder. *Behavioral and Brain Functions*.

[35] Kieling C., Genro J. P., Hutz M. H., Rohde L. A. (2008). The− 1021 C/T DBH polymorphism is associated with neuropsychological performance among children and adolescents with ADHD. *American Journal of Medical Genetics Part B: Neuropsychiatric Genetics*.

[36] Rajan R., Krishnan S., Sarma G., Sarma S. P., Kishore A. (2018). Dopamine receptor D3 rs6280 is associated with aberrant decision-making in Parkinson’s disease. *Movement Disorders Clinical Practice*.

[37] Robbins T. (2002). The 5-choice serial reaction time task: behavioural pharmacology and functional neurochemistry. *Psychopharmacology*.

[38] Logan G. D., Van Zandt T., Verbruggen F., Wagenmakers E.-J. (2014). On the ability to inhibit thought and action: general and special theories of an act of control. *Psychological Review*.

[39] McDonald V., Hauner K. K., Chau A., Krueger F., Grafman J. (2017). Networks underlying trait impulsivity: evidence from voxel-based lesion-symptom mapping. *Human Brain Mapping*.

[40] Patton J. H., Stanford M. S., Barratt E. S. (1995). Factor structure of the Barratt impulsiveness scale. *Journal of Clinical Psychology*.

[41] Stanford M. S., Mathias C. W., Dougherty D. M., Lake S. L., Anderson N. E., Patton J. H. (2009). Fifty years of the Barratt impulsiveness scale: an update and review. *Personality and Individual Differences*.

[42] Vasconcelos A. G., Malloy-Diniz L., Correa H. (2012). Systematic review of psychometric proprieties of Barratt impulsiveness scale version 11 (BIS-11). *Clinical Neuropsychiatry: Journal of Treatment Evaluation*.

[43] Weintraub D., Hoops S., Shea J. A. (2009). Validation of the questionnaire for impulsive-compulsive disorders in Parkinson’s disease. *Movement Disorders*.

[44] Papay K., Mamikonyan E., Siderowf A. D. (2011). Patient versus informant reporting of ICD symptoms in Parkinson’s disease using the QUIP: validity and variability. *Parkinsonism & Related Disorders*.

[45] Evans A. H., Okai D., Weintraub D. (2019). Scales to assess impulsive and compulsive behaviors in Parkinson’s disease: critique and recommendations. *Movement Disorders*.

[46] Anderton R. S., Byrnes M., Jefferson A. (2017). Sleep disturbance and serum ferritin levels associate with high impulsivity and impulse control disorders in male Parkinson’s disease patients. *American Journal of Psychiatry and Neuroscience*.

[47] Fantini M. L., Durif F., Marques A. (2019). Impulse control disorders in REM sleep behavior disorder. *Current Treatment Options in Neurology*.

[48] Association A. P. (2013). *Diagnostic and Statistical Manual of Mental Disorders (DSM-5®)*.

[49] Beier K., Meinck H. M., Berger C., Mehrhoff F. (2003). Sexuelle Delinquenz und Morbus Parkinson. *Nervenarzt, Der*.

[50] Voon V., Potenza M. N., Thomsen T. (2007). Medication-related impulse control and repetitive behaviors in Parkinson’s disease. *Current Opinion in Neurology*.

[51] Leroi I., Harbishettar V., Andrews M., McDonald K., Byrne E. J., Burns A. (2012). Carer burden in apathy and impulse control disorders in Parkinson’s disease. *International Journal of Geriatric Psychiatry*.

[52] Phu A. L., Xu Z., Brakoulias V. (2014). Effect of impulse control disorders on disability and quality of life in Parkinson’s disease patients. *Journal of Clinical Neuroscience*.

[53] Voon V., Fox S. H. (2007). Medication-related impulse control and repetitive behaviors in Parkinson disease. *Archives of Neurology*.

[54] Avanzi M., Baratti M., Cabrini S., Uber E., Brighetti G., Bonfà F. (2006). Prevalence of pathological gambling in patients with Parkinson’s disease. *Movement Disorders*.

[55] Giladi N., Weitzman N., Schreiber S., Shabtai H., Peretz C. (2007). New onset heightened interest or drive for gambling, shopping, eating or sexual activity in patients with Parkinson’s disease: the role of dopamine agonist treatment and age at motor symptoms onset. *Journal of Psychopharmacology*.

[56] Weintraub D., Koester J., Potenza M. N. (2010). Impulse control disorders in Parkinson disease: a cross-sectional study of 3090 patients. *Archives of Neurology*.

[57] Bastiaens J., Dorfman B. J., Christos P. J., Nirenberg M. J. (2013). Prospective cohort study of impulse control disorders in Parkinson’s disease. *Movement Disorders*.

[58] Corvol J. C., Artaud F., Cormier-Dequaire F. (2018). Longitudinal analysis of impulse control disorders in Parkinson disease. *Neurology*.

[59] Ramirez-Zamora A., Gee L., Boyd J., Biller J. (2016). Treatment of impulse control disorders in Parkinson’s disease: practical considerations and future directions. *Expert Review of Neurotherapeutics*.

[60] Okai D., Askey-Jones S., Samuel M. (2013). Trial of CBT for impulse control behaviors affecting Parkinson patients and their caregivers. *Neurology*.

[61] Fasano A., Pettorruso M., Ricciardi L., Conte G., Bentivoglio A. R. (2010). Punding in Parkinson’s disease: the impact of patient’s awareness on diagnosis. *Movement Disorders*.

[62] Gazzaley A., D’Esposito M. (2007). Unifying prefrontal cortex function: executive control, neural networks, and top-down modulation. *The Human Frontal Lobes: Functions and Disorders*.

[63] Bezdjian S., Baker L. A., Tuvblad C. (2011). Genetic and environmental influences on impulsivity: a meta-analysis of twin, family and adoption studies. *Clinical Psychology Review*.

[64] Balleine B. W., Delgado M. R., Hikosaka O. (2007). The role of the dorsal striatum in reward and decision-making: figure 1. *Journal of Neuroscience*.

[65] Eagle D., Robbins T. (2003). Inhibitory control in rats performing a stop-signal reaction-time task: effects of lesions of the medial striatum and d-amphetamine. *Behavioral Neuroscience*.

[66] Robertson C. L., Ishibashi K., Mandelkern M. A. (2015). Striatal D1-and D2-type dopamine receptors are linked to motor response inhibition in human subjects. *Journal of Neuroscience*.

[67] Tedford S. E., Persons A. L., Napier T. C. (2015). Dopaminergic lesions of the dorsolateral striatum in rats increase delay discounting in an impulsive choice task. *PLoS One*.

[68] Meredith G. E., Baldo B. A., Andrezjewski M. E., Kelley A. E. (2008). The structural basis for mapping behavior onto the ventral striatum and its subdivisions. *Brain Structure and Function*.

[69] Cole B. J., Robbins T. W. (1989). Effects of 6-hydroxydopamine lesions of the nucleus accumbens septi on performance of a 5-choice serial reaction time task in rats: implications for theories of selective attention and arousal. *Behavioural Brain Research*.

[70] Dalley J. W., Fryer T. D., Brichard L. (2007). Nucleus accumbens D2/3 receptors predict trait impulsivity and cocaine reinforcement. *Science*.

[71] Basar K., Sesia T., Groenewegen H., Steinbusch H. W., Visser-Vandewalle V., Temel Y. (2010). Nucleus accumbens and impulsivity. *Progress in Neurobiology*.

[72] Cardinal R. N., Pennicott D. R., Lakmali C., Robbins T. W., Robbins T. W., Everitt B. J. (2001). Impulsive choice induced in rats by lesions of the nucleus accumbens core. *Science*.

[73] Besson M., Belin D., McNamara R. (2010). Dissociable control of impulsivity in rats by dopamine D2/3 receptors in the core and shell subregions of the nucleus accumbens. *Neuropsychopharmacology*.

[74] Zaichenko M. I., Merzhanova G. K. (2014). The effects of dopamine D1/D2 receptor agonists and blockers on the behavior of rats with different choices of reinforcement value. *Neuroscience and Behavioral Physiology*.

[75] Alexander G. E., DeLong M. R., Strick P. L. (1986). Parallel organization of functionally segregated circuits linking basal ganglia and cortex. *Annual Review of Neuroscience*.

[76] Sellitto M., Ciaramelli E., di Pellegrino G. (2010). Myopic discounting of future rewards after medial orbitofrontal damage in humans. *Journal of Neuroscience*.

[77] Lee A. K., Jerram M., Fulwiler C., Gansler D. A. (2011). Neural correlates of impulsivity factors in psychiatric patients and healthy volunteers: a voxel-based morphometry study. *Brain Imaging and Behavior*.

[78] Matsuo K., Nicoletti M., Nemoto K. (2009). A voxel‐based morphometry study of frontal gray matter correlates of impulsivity. *Human Brain Mapping*.

[79] Schiffer B., Müller B. W., Scherbaum N. (2010). Impulsivity-related brain volume deficits in schizophrenia-addiction comorbidity. *Brain*.

[80] Alvarez J. A., Emory E. (2006). Executive function and the frontal lobes: a meta-analytic review. *Neuropsychology Review*.

[81] McClure S. M., Ericson K. M., Laibson D. I., Loewenstein G., Cohen J. D. (2007). Time discounting for primary rewards. *Journal of Neuroscience*.

[82] McClure S. M., Laibson D. I., Loewenstein G., Cohen J. D. (2004). Separate neural systems value immediate and delayed monetary rewards. *Science*.

[83] Hare T. A., Camerer C. F., Rangel A. (2009). Self-control in decision-making involves modulation of the vmPFC valuation system. *Science*.

[84] Aron A. R., Robbins T. W., Poldrack R. A. (2014). Inhibition and the right inferior frontal cortex: one decade on. *Trends in Cognitive Sciences*.

[85] Fonken Y. M., Rieger J. W., Tzvi E. (2016). Frontal and motor cortex contributions to response inhibition: evidence from electrocorticography. *Journal of Neurophysiology*.

[86] Jacobson L., Javitt D. C., Lavidor M. (2011). Activation of inhibition: diminishing impulsive behavior by direct current stimulation over the inferior frontal gyrus. *Journal of Cognitive Neuroscience*.

[87] Swick D., Ashley V., Turken A. U. (2008). Left inferior frontal gyrus is critical for response inhibition. *BMC Neuroscience*.

[88] Noble E. E., Wang Z., Liu C. M. (2019). Hypothalamus-hippocampus circuitry regulates impulsivity via melanin-concentrating hormone. *Nature Communications*.

[89] Coccaro E. F., Lee R., McCloskey M., Csernansky J. G., Wang L. (2015). Morphometric analysis of amygdla and hippocampus shape in impulsively aggressive and healthy control subjects. *Journal of Psychiatric Research*.

[90] Kumari V., Barkataki I., Goswami S., Flora S., Das M., Taylor P. (2009). Dysfunctional, but not functional, impulsivity is associated with a history of seriously violent behaviour and reduced orbitofrontal and hippocampal volumes in schizophrenia. *Psychiatry Research: Neuroimaging*.

[91] Sala M., Caverzasi E., Lazzaretti M. (2011). Dorsolateral prefrontal cortex and hippocampus sustain impulsivity and aggressiveness in borderline personality disorder. *Journal of Affective Disorders*.

[92] Dalley J. W., Everitt B. J., Robbins T. W. (2011). Impulsivity, compulsivity, and top-down cognitive control. *Neuron*.

[93] Pattij T., Vanderschuren L. J. (2008). The neuropharmacology of impulsive behaviour. *Trends in Pharmacological Sciences*.

[94] Dahlström A., Fuxe K., Olson L., Ungerstedt U. (1964). Ascending systems of catecholamine neurons from the lower brain stem. *Acta Physiologica Scandinavica*.

[95] Beliveau V., Svarer C., Frokjaer V. G., Knudsen G. M., Greve D. N., Fisher P. M. (2015). Functional connectivity of the dorsal and median raphe nuclei at rest. *NeuroImage*.

[96] Schwarz L. A., Luo L. (2015). Organization of the locus coeruleus-norepinephrine system. *Current Biology*.

[97] Jupp B., Dalley J. W. (2016). The genetics of impulsivity: a synthesis of findings in humans and rodent models. *Animal Models of Behavior Genetics*.

[98] Buckholtz J. W., Treadway M. T., Cowan R. L. (2010). Dopaminergic network differences in human impulsivity. *Science*.

[99] Clark L., Stokes P. R., Wu K. (2012). Striatal dopamine D2/D3 receptor binding in pathological gambling is correlated with mood-related impulsivity. *NeuroImage*.

[100] Eagle D. M., Wong J. C., Allan M. E., Mar A. C., Theobald D. E., Robbins T. W. (2011). Contrasting roles for dopamine D1 and D2 receptor subtypes in the dorsomedial striatum but not the nucleus accumbens core during behavioral inhibition in the stop-signal task in rats. *Journal of Neuroscience*.

[101] Reeves S. J., Polling C., Stokes P. R. (2012). Limbic striatal dopamine D2/3 receptor availability is associated with non-planning impulsivity in healthy adults after exclusion of potential dissimulators. *Psychiatry Research: Neuroimaging*.

[102] Drew D. S., Muhammed K., Baig F. (2020). Dopamine and reward hypersensitivity in Parkinson’s disease with impulse control disorder. *Brain*.

[103] Harrison A. A., Everitt B. J., Robbins T. W. (1997). Central 5-HT depletion enhances impulsive responding without affecting the accuracy of attentional performance: interactions with dopaminergic mechanisms. *Psychopharmacology*.

[104] Walderhaug E., Lunde H., Nordvik J. E., Landrø N., Refsum H., Magnusson A. (2002). Lowering of serotonin by rapid tryptophan depletion increases impulsiveness in normal individuals. *Psychopharmacology*.

[105] Lindström M. B., Ryding E., Bosson P., Ahnlide J.-A., Rosén I., Träskman-Bendz L. (2004). Impulsivity related to brain serotonin transporter binding capacity in suicide attempters. *European Neuropsychopharmacology*.

[106] Mann J. J., Brent D. A., Arango V. (2001). The neurobiology and genetics of suicide and attempted suicide: a focus on the serotonergic system. *Neuropsychopharmacology*.

[107] Linnoila V. M., Virkkunen M. (1992). Aggression, suicidality, and serotonin. *The Journal of Clinical Psychiatry*.

[108] De Simoni M. G., Dal Toso G., Fodritto F., Sokola A., Algeri S. (1987). Modulation of striatal dopamine metabolism by the activity of dorsal raphe serotonergic afferences. *Brain Research*.

[109] Baarendse P. J., Winstanley C. A., Vanderschuren L. J. (2013). Simultaneous blockade of dopamine and noradrenaline reuptake promotes disadvantageous decision making in a rat gambling task. *Psychopharmacology (Berl)*.

[110] Vriend C. (2018). The neurobiology of impulse control disorders in Parkinson’s disease: from neurotransmitters to neural networks. *Cell and Tissue Research*.

[111] Robinson E. S. J., Eagle D. M., Mar A. C. (2007). Similar effects of the selective noradrenaline reuptake inhibitor atomoxetine on three distinct forms of impulsivity in the rat. *Neuropsychopharmacology*.

[112] Faraone S. V., Biederman J., Spencer T. (2005). Efficacy of atomoxetine in adult attention-Deficit/Hyperactivity Disorder: a drug-placebo response curve analysis. *Behavioral and Brain Functions*.

[113] del Campo N., Chamberlain S. R., Sahakian B. J., Robbins T. W. (2011). The roles of dopamine and noradrenaline in the pathophysiology and treatment of attention-deficit/hyperactivity disorder. *Biological Psychiatry*.

[114] Hesse S., Müller U., Rullmann M. (2017). The association between in vivo central noradrenaline transporter availability and trait impulsivity. *Psychiatry Research: Neuroimaging*.

[115] Lee J. Y., Kim J. M., Kim J. W. (2010). Association between the dose of dopaminergic medication and the behavioral disturbances in Parkinson disease. *Parkinsonism & Related Disorders*.

[116] Sohtaoğlu M., Demiray D. Y., Kenangil G., Ozekmekçi S., Erginöz E. (2010). Long term follow-up of Parkinson’s disease patients with impulse control disorders. *Parkinsonism & Related Disorders*.

[117] Marín-Lahoz J., Pagonabarraga J., Martinez-Horta S. (2018). Parkinson’s disease: impulsivity does not cause impulse control disorders but boosts their severity. *Frontiers in Psychiatry*.

[118] Cornelius J. R., Tippmann-Peikert M., Slocumb N. L., Frerichs C. F., Silber M. H. (2010). Impulse control disorders with the use of dopaminergic agents in restless legs syndrome: a case-control study. *Sleep*.

[119] Holman A. J. (2009). Impulse control disorder behaviors associated with pramipexole used to treat fibromyalgia. *Journal of Gambling Studies*.

[120] Antonini A., Barone P., Bonuccelli U. (2017). ICARUS study: prevalence and clinical features of impulse control disorders in Parkinson’s disease. *Journal of Neurology Neurosurgery and Psychiatry*.

[121] Moore T. J., Glenmullen J., Mattison D. R. (2014). Reports of pathological gambling, hypersexuality, and compulsive shopping associated with dopamine receptor agonist drugs. *JAMA Internal Medicine*.

[122] Valença G. T., Glass P. G., Negreiros N. N. (2013). Past smoking and current dopamine agonist use show an independent and dose-dependent association with impulse control disorders in Parkinson’s disease. *Parkinsonism & Related Disorders*.

[123] Vela L., Martínez Castrillo J. C., García Ruiz P. (2016). The high prevalence of impulse control behaviors in patients with early-onset Parkinson’s disease: a cross-sectional multicenter study. *Journal of the Neurological Sciences*.

[124] Poletti M., Bonuccelli U. (2013). Acute and chronic cognitive effects of levodopa and dopamine agonists on patients with Parkinson’s disease: a review. *Therapeutic Advances in Psychopharmacology*.

[125] Kish S. J., Shannak K., Hornykiewicz O. (1988). Uneven pattern of dopamine loss in the striatum of patients with idiopathic Parkinson’s disease. *New England Journal of Medicine*.

[126] Evans A. H., Lawrence A. D., Potts J., Appel S., Lees A. J. (2005). Factors influencing susceptibility to compulsive dopaminergic drug use in Parkinson disease. *Neurology*.

[127] Murray A. M., Ryoo H. L., Gurevich E., Joyce J. N. (1994). Localization of dopamine D3 receptors to mesolimbic and D2 receptors to mesostriatal regions of human forebrain. *Proceedings of the National Academy of Sciences*.

[128] Evans A. H., Pavese N., Lawrence A. D. (2006). Compulsive drug use linked to sensitized ventral striatal dopamine transmission. *Annals of Neurology*.

[129] Stark A. J., Smith C. T., Lin Y. C. (2018). Nigrostriatal and mesolimbic D(2/3) receptor expression in Parkinson’s disease patients with compulsive reward-driven behaviors. *Journal of Neuroscience*.

[130] Tabor A., Möller D., Hübner H., Kornhuber J., Gmeiner P. (2017). Visualization of ligand-induced dopamine D2S and D2L receptor internalization by TIRF microscopy. *Scientific Reports*.

[131] Bernosky-Smith K. A., Qiu Y. Y., Feja M. (2018). Ventral tegmental area D2 receptor knockdown enhances choice impulsivity in a delay-discounting task in rats. *Behavioural Brain Research*.

[132] Hisahara S., Shimohama S. (2011). Dopamine receptors and Parkinson’s disease. *International Journal of Medicinal Chemistry*.

[133] van Oosten R. V., Verheij M. M. M., Cools A. R. (2005). Bilateral nigral 6-hydroxydopamine lesions increase the amount of extracellular dopamine in the nucleus accumbens. *Experimental Neurology*.

[134] Boileau I., Guttman M., Rusjan P. (2009). Decreased binding of the D3 dopamine receptor-preferring ligand [11C]-(+)-PHNO in drug-naive Parkinson’s disease. *Brain*.

[135] Payer D. E., Guttman M., Kish S. J. (2015). [11C]‐(+)‐PHNO PET imaging of dopamine D2/3 receptors in Parkinson’s disease with impulse control disorders. *Movement Disorders*.

[136] Dickson D. W. (2012). Parkinson’s disease and parkinsonism: neuropathology. *Cold Spring Harbor Perspectives in Medicine*.

[137] Halliday G. M., Blumbergs P. C., Cotton R. G. H., Blessing W. W., Geffen L. B. (1990). Loss of brainstem serotonin- and substance P-containing neurons in Parkinson’s disease. *Brain Research*.

[138] Kish S. J., Tong J., Hornykiewicz O. (2008). Preferential loss of serotonin markers in caudate versus putamen in Parkinson’s disease. *Brain*.

[139] Kordower J. H., Olanow C. W., Dodiya H. B. (2013). Disease duration and the integrity of the nigrostriatal system in Parkinson’s disease. *Brain*.

[140] Mantovani E., Martini A., Tamburin S. (2023). Pharmacological and non‐pharmacological treatments for impulsive‐compulsive behaviors in Parkinson’s disease. *Cochrane Database of Systematic Reviews*.

